# Progress Toward Elimination of Onchocerciasis in the Americas — 1993–2012

**Published:** 2013-05-24

**Authors:** National onchocerciasis elimination programs of Brazil, Colombia, Ecuador, Guatemala, Mexico, and Venezuela, Onchocerciasis Elimination Program for the Americas/The Carter Center, Guatemala City, Guatemala, Pan American Health Organization, Div of Parasitic Diseases and Malaria, Center for Global Health, CDC

Onchocerciasis (river blindness) is caused by the parasitic worm *Onchocerca volvulus*, transmitted to humans by the bite of infected black flies of the genus *Simulium*, and is characterized by chronic skin disease, severe itching, and eye lesions that can progress to complete blindness. Currently, among approximately 123 million persons at risk for infection in 38 endemic countries, at least 25.7 million are infected, and 1 million are blinded or have severe visual impairment (1). Periodic, communitywide mass drug administration (MDA) with ivermectin (Mectizan, Merck) prevents eye and skin disease and might interrupt transmission of the infection, depending on the coverage, duration, and frequency of MDA. The Onchocerciasis Elimination Program for the Americas (OEPA) was launched in response to a 1991 resolution of the Pan American Health Organization (PAHO) calling for the elimination of onchocerciasis from the Americas. By the end of 2012, transmission of the infection, judged by surveys following World Health Organization (WHO) guidelines, had been interrupted or eliminated in four of the six endemic countries in the WHO Americas Region. Thus, in 2013, only 4% (23,378) of the 560,911 persons originally at risk in the Americas will be under ivermectin MDA. Active transmission currently is limited to two foci among Yanomami indigenes in adjacent border areas of Venezuela and Brazil.

In 2001, WHO established a set of technical guidelines to help onchocerciasis programs determine whether interruption of transmission has occurred and whether MDA with ivermectin could be stopped (2,3). The process includes three key phases: 1) suppression of transmission, when infective-stage larvae are no longer introduced into the human population by the vectors, but the parasite population in the human reservoir maintains the ability to recover if treatments are withdrawn; 2) interruption of transmission, when the parasite population is thought to be unable to recover and treatments can be halted; and 3) elimination of transmission, when a posttreatment surveillance period of at least 3 years confirms that the parasite population has not recovered in the absence of interventions (4). Ocular morbidity is considered eliminated when the prevalence of acute eye lesions attributable to onchocerciasis falls below 1% (3). When all the foci in a country reach the elimination stage, final country verification can be considered by an independent international team of experts convened under the auspices of WHO.

OEPA* was launched in response to a 1991 PAHO resolution that called for the elimination of onchocerciasis morbidity from the Americas by 2007 (5). In 2008, based on significant OEPA achievements, PAHO and its member states renewed the call to eliminate onchocerciasis throughout the region and set a goal to interrupt transmission of the parasite throughout the region by 2012.† A PAHO resolution in 2009 that calls for the elimination or control of 12 neglected, poverty-related infectious diseases in the Americas by 2015 includes onchocerciasis as one of its elimination targets.§

The primary strategy for eliminating onchocerciasis from the Americas has been ivermectin MDA every 6 months, with health education and community mobilization, in all affected communities of the 13 endemic foci in the six affected countries (Figure) (5,6). MDA aims to achieve at least 85% coverage of the population at risk and eligible for treatment. Communities targeted for MDA are divided by baseline onchocerciasis prevalence into hyperendemic (≥60%), mesoendemic (≥20%, but <60%), and hypoendemic (evidence of autochthonous cases, but with prevalence <20%). Transmission is most difficult to break in hyperendemic areas, where MDA might need to be given every 3 months (7).

A total of 11,069,285 MDA ivermectin treatments were administered in the Americas during 1993–2012. By the end of 2012, transmission of the infection, as judged by surveys following established guidelines, had been interrupted or eliminated in four of the six countries, and ivermectin MDAs were halted in 11 of the 13 foci, with active transmission occurring only in two foci among Yanomami indigenous populations in adjacent border areas of southern Venezuela and northern Brazil. In 2013, only 4% (23,378) of the 560,911 persons originally at risk in the Americas will be targeted for ivermectin MDA. Ocular morbidity was detected only in southern Venezuela (Table). Since 1995, no new blindness has been attributed to onchocerciasis in the Americas.

## Country Reports

### Venezuela

The Northcentral, Northeast, and South foci in Venezuela comprised 119,358 persons at risk for onchocerciasis infection, the third highest national total in the Americas. The South focus in Venezuela had the second highest rate of microfilariae measured in the skin at baseline among the 13 foci in the Americas (Table). Venezuela has conducted MDA semiannually in 100 hyperendemic, 212 mesoendemic, and 297 hypoendemic communities, beginning in 2000. In 2010, the program began conducting MDA quarterly in 66 hyperendemic communities in the South and Northeastern foci, eventually extending this to an additional 35 hyperendemic and five mesoendemic communities. When transmission was interrupted in the Northcentral and Northeast foci in 2010 and 2012, respectively, programs in those two foci had administered 17 and 20 rounds of mass treatment, with reported coverage of ≥85%. In 2013, treatments will be halted in the Northeast focus. The main challenges for the South focus (which had completed 14 rounds of MDA during 2006–2012) now are to search the remaining suspect areas for any still-unidentified endemic Yanomami communities and immediately increase MDA frequency to quarterly in all hyperendemic villages.

### Brazil

The single focus of onchocerciasis in Brazil is among the Yanomami population living in an area contiguous with the endemic focus of South in Venezuela. Brazil’s focus includes 12,988 persons in 22 endemic administrative areas (seven hyperendemic, nine mesoendemic, and six hypoendemic) called “polos bases.” As in Venezuela, the affected area is remote and densely forested, and the migratory Yanomami move across the border at will. The Brazilian program administered 24 semiannual MDAs with at least 85% coverage during 2001–2012. The program began administering MDA treatments quarterly to seven hyperendemic and three mesoendemic polo bases in 2011. The latest surveys suggest that Brazil is close to suppressing onchocerciasis transmission in its part of the shared Yanomami area.

### Guatemala

With four foci and 231,467 persons at risk, Guatemala had the greatest number of persons at risk for onchocerciasis in the Americas. The four foci encompass a total of 518 endemic communities (42 hyperendemic, 15 mesoendemic, and 461 hypoendemic). During 2001–2011, Guatemala conducted MDA and health education semiannually, achieving a reported 21 rounds of coverage of ≥85%. In 2006 and 2007, respectively, Guatemala’s Santa Rosa and Escuintla foci were the first in the region to interrupt transmission in the Americas, (Table), followed by the Huehuetenango focus in 2008. MDA ended in Guatemala with cessation of treatment in the Central focus in 2012.

### Mexico

The second-highest number of persons at risk for onchocerciasis (169,869) in the Americas were in three foci and 670 communities (39 hyperendemic, 220 mesoendemic, and 411 hypoendemic) in Mexico (Table). Mexico has achieved 25 consecutive rounds of MDA with coverage of ≥85% during 2001–2011. In 2003, Mexico began quarterly MDA in 37 hyperendemic communities in the largest of its foci (South Chiapas) in an effort to accelerate interruption of transmission, becoming the first country to adopt this innovation. North Chiapas became the third focus to interrupt transmission in the Americas and Oaxaca was the sixth. MDA ended in Mexico with cessation of treatment in South Chiapas in 2012.

### Ecuador

The single focus of onchocerciasis in Ecuador includes 119 communities (42 hyperendemic, 23 mesoendemic, and 54 hypoendemic) distributed among three river valleys in the Province of Esmeraldas. Although Ecuador’s population at risk for onchocerciasis was relatively small (25,863), this focus had the highest prevalence of microfilariae in the skin at baseline of the 13 American foci. One of the two black fly vectors here, *Simulium exiguum*, is one of the most efficient transmitters of onchocerciasis in the Americas, comparable to *Simulium damnosum*, the major vector in Africa. Ecuador completed 23 MDA semiannual rounds of ≥85% coverage before interrupting transmission in 2009 and halting MDA in 2010. Posttreatment surveillance was completed successfully throughout the country in 2012. In 2013, Ecuador should become the second country in the Americas to request verification of elimination of onchocerciasis from WHO.

### Colombia

The single focus of onchocerciasis in Colombia was a mesoendemic community. Colombia conducted 20 rounds of MDA coverage of at least 85% before it interrupted transmission in 2007 and halted MDA in 2008. Colombia successfully completed posttreatment surveillance in 2010, and applied to WHO for verification of elimination of onchocerciasis in 2012 (7).

What is already known on this topic?

In 1991, the Pan American Health Organization called for the elimination of onchocerciasis (river blindness) transmission in the Americas. Since then, the population under mass drug treatment in the Americas for onchocerciasis has been decreasing each year, from an estimated 500,000 to approximately 23,000.

What is added by this report?

Transmission of *Onchocerca volvulus* has been interrupted in 11 of the 13 foci in the Americas, leaving only 4% of the previous at risk population still needing continued mass drug administration. Colombia, Ecuador, Guatemala, and Mexico have all interrupted transmission. Transmission continues among the Yanomami indigenes in the Amazonian forest area on the border between Brazil and Venezuela.

What are the implications for public health practice?

Although earlier target dates of 2007 and 2012 for elimination of onchocerciasis in the Americas were missed, progress is accelerating, and elimination is likely within the next few years. Success in the final transmission zone will require intensified efforts and cross-border collaboration. Preliminary results from the Brazilian side are encouraging and indicate that transmission also can be interrupted in this region. Successful elimination of onchocerciasis in the Americas has and will continue to provide strong impetus and lessons learned for pursuing elimination of onchocerciasis in Africa.

### Editorial Note

By the end of 2012, *O. volvulus* transmission was interrupted or eliminated in 11 of the 13 foci in the Americas. The current OEPA goal, under PAHO Resolution CD49.R19, is to interrupt transmission throughout the Americas by 2015. The challenges, therefore, are the two remaining endemic crossborder foci of Amazonas in Brazil and South in Venezuela. These are, in fact, a single epidemiologic unit that needs to be addressed through closely coordinated activities by the two countries. To accelerate the elimination process, the OEPA strategy is to increase ivermectin MDA to quarterly administration in the most highly endemic communities alongside the border, and identify and intensively treat any as yet unknown endemic communities.

The OEPA program is distinguished by the substantial proportion (38%) of its costs (approximately $121 million over the past 2 decades, which includes the value of the donated medicines) that was contributed by the six endemic countries. This was supplemented by critical support from external partners. The program also has benefited from its strong emphasis on data-driven decision processes, strong community mobilization, and innovative health education methods.¶ OEPA’s achievements have encouraged reorientation of onchocerciasis goals in the disease’s main stronghold (Africa) from morbidity control to transmission elimination.

## Figures and Tables

**FIGURE:**
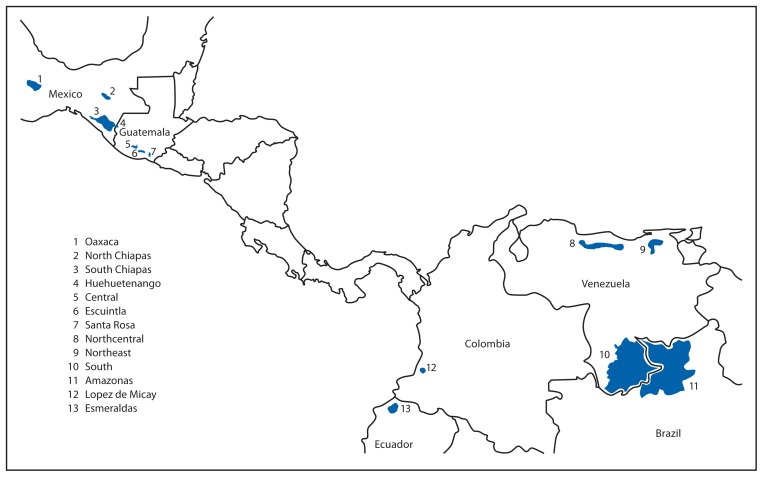
Thirteen onchocerciasis foci — World Health Organization Region of the Americas, 2005

**TABLE:** Baseline indices and current transmission status of onchocerciasis — 13 foci, World Health Organization Region of the Americas, 1979–2012

Identifier*	Focus area	Population at risk	Vector (*Simulium*)	Baseline indices				Transmission and ocular morbidity status		

				Mf in skin		MfAC				
		
				(%)	Year	(%)	Year	Interrupted	Eliminated	Ongoing
1	Oaxaca, Mexico	44,919	*S. ochraceum*	(7.3)	1983	(0)	1995	2008	2011	
2	North Chiapas, Mexico	7,125	*S. ochraceum*	(1.5)	1995	(0.6)	1995	2007	2010	
3	South Chiapas, Mexico	117,825	*S. ochraceum*	(14.5)	1995	(1.5)	1995	2011		
4	Huehuetenango, Guatemala	30,239	*S. ochraceum*	(2.9)	1987	(7.2)	1981	2008	2011	
5	Central, Guatemala	126,430	*S. ochraceum*	(52.2)	1994	(20.7)	1981	2011		
6	Escuintla, Guatemala	62,590	*S. ochraceum*	(29.5)	1979	(6.2)	1979	2007	2010	
7	Santa Rosa, Guatemala	12,208	*S. ochraceum*	(3.0)	1983	NA	—	2006	2010	
8	Northcentral, Venezuela	14,385	*S. metallicum*	(44.3)	1999	(31.0)	1999	2010		
9	Northeast, Venezuela	94,583	*S. metallicum*	(28.0)	1999	(21.7)	1999	2012		
10	South, Venezuela	10,390	*S. guianense* *S. oyapockense*	(75.0)	1998	(10.5)	1998			Ongoing†
11	Amazonas, Brazil	12,988	*S. guianense* *S. oyapockense* *S. incrustatum*	(63.3)	1995	(31.2)	1995			Ongoing§
12	Lopez de Micay, Colombia	1,366	*S. exiguum*	(39.6)	1995	(0)	1996	2007	2010	
13	Esmeraldas, Ecuador	25,863	*S. exiguum* *S. quadrivittatum*	(78.7)	1991	(24.7)	1991	2009	2012¶	
	**Total (Mean)**	**560,911**		**(33.8)**		**(12.9)**				

**Abbreviations:** NA = not available; Mf = microfilariae; MfAC = microfilariae in anterior chamber of the eye.

* Matches numbers shown on map in Figure.

† Only focus with demonstrable ocular morbidity.

§ Possibly suppressed.

¶ Pending review by Ecuador Ministry of Health.

## Footnotes

* Additional information available at http://www.oepa.net.

† Resolution CD48.R12. Towards the elimination of onchocerciasis (river blindness) in the Americas. Available at http://www1.paho.org/english/gov/cd/cd48.r12-e.pdf.

§ Resolution CD49.R19. Elimination of neglected diseases and other poverty-related infections. Available at http://new.paho.org/hq/dmdocuments/2009/CD49.R19%20(Eng.).pdf.

¶ Additional information is available at http://new.paho.org/blogs/arteysalud.

## References

[1] Crump A, Morel CM, Omura S (2012). The onchocerciasis chronicle: from the beginning to the end?. Trends Parasitol.

[2] World Health Organization (2001). Certification of elimination of human onchocerciasis: criteria and procedures.

[3] Lindblade KA, Arana B, Zea-Flores G (2007). Elimination of *Onchocerca volvulus* transmission in the Santa Rosa focus of Guatemala. Am J Trop Med Hyg.

[4] Program Coordinating Committee and OEPA staff (2012). Guide to detecting a potential recrudescence of onchocerciasis during the posttreatment surveillance period: the American paradigm. Res Rep Trop Med.

[5] Blanks J, Richards F, Beltran F (1998). The Onchocerciasis Elimination Program of the Americas: a history of partnership. Rev Panam Salud Publica.

[6] Sauerbrey M (2008). The Onchocerciasis Elimination Program for the Americas (OEPA). Ann Trop Med Parasitol.

[7] World Health Organization (2012). InterAmerican Conference on Onchocerciasis, 2011: interruption of transmission in Guatemala and Mexico. Wkly Epidemiol Rec.

